# ProSAAS-Derived Peptides Are Differentially Processed and Sorted in Mouse Brain and AtT-20 Cells

**DOI:** 10.1371/journal.pone.0104232

**Published:** 2014-08-22

**Authors:** Jonathan H. Wardman, Lloyd D. Fricker

**Affiliations:** 1 Department of Molecular Pharmacology, Albert Einstein College of Medicine, Bronx, New York, United States of America; 2 Dominick P. Purpura Department of Neuroscience, Albert Einstein College of Medicine, Bronx, New York, United States of America; University of Rouen, France

## Abstract

ProSAAS is the precursor for some of the most abundant peptides found in mouse brain and other tissues, including peptides named SAAS, PEN, and LEN. Both SAAS and LEN are found in big and little forms due to differential processing. Initial processing of proSAAS is mediated by furin (and/or furin-like enzymes) and carboxypeptidase D, while the smaller forms are generated by secretory granule prohormone convertases and carboxypeptidase E. In mouse hypothalamus, PEN and big LEN colocalize with neuropeptide Y. In the present study, little LEN and SAAS were detected in mouse hypothalamus but not in cell bodies of neuropeptide Y-expressing neurons. PEN and big LEN show substantial colocalization in hypothalamus, but big LEN and little LEN do not. An antiserum to SAAS that detects both big and little forms of this peptide did not show substantial colocalization with PEN or big LEN. To further study this, the AtT-20 cells mouse pituitary corticotrophic cell line was transfected with rat proSAAS and the distribution of peptides examined. As found in mouse hypothalamus, only some of the proSAAS-derived peptides colocalized with each other in AtT-20 cells. The two sites within proSAAS that are known to be efficiently cleaved by furin were altered by site-directed mutagenesis to convert the P4 Arg into Lys; this change converts the sequences from furin consensus sites into prohormone convertase consensus sites. Upon expression of the mutated form of proSAAS in AtT-20 cells, there was significantly more colocalization of proSAAS-derived peptides PEN and SAAS. Taken together, these results indicate that proSAAS is initially cleaved in the Golgi or trans-Golgi network by furin and/or furin-like enzymes and the resulting fragments are sorted into distinct vesicles and further processed by additional enzymes into the mature peptides.

## Introduction

Most peptide hormones and neuropeptides are produced by the selective cleavage of precursor proteins within the secretory pathway [1]. The cleavages occur at specific sites, often involving pairs of basic residues such as Lys-Arg or Arg-Arg [2]. In some cases, the pair of basic residues is separated by 2, 4, or 6 other amino acids. Less frequently, cleavage can occur at monobasic sites or at sites lacking basic residues. The major endopeptidases responsible for cleavages at basic sites include the trans-Golgi network (TGN) enzyme furin, and two secretory vesicle enzymes: prohormone convertase 1/3 (PC1/3) and prohormone convertase 2 (PC2) [2]–[5]. Other TGN furin-like endopeptidases include proprotein convertase 7 and proprotein convertase 5/6B [6], [7]. All of these endopeptidases cleave to the C-terminal side of the basic amino acid in the consensus site, producing peptide processing intermediates that contain C-terminal Lys and/or Arg residues [4], [5], [8]. These C-terminal basic residues are removed by carboxypeptidase D (CPD) in the TGN, or by carboxypeptidase E (CPE) in the secretory vesicles [9], [10]. The effectiveness of each of these endo- and carboxypeptidases is controlled by the conditions inside the vesicles in which the peptides reside, including the intravesicular pH and calcium ion concentration, and the amino acid sequences of the cleavage sites [1], [2]. Processing of peptides is therefore affected by the intrinsic properties of the peptides themselves, the properties of their processing enzymes, and the environment in which they are processed.

Various tissues may express a particular neuropeptide precursor but yield different cleavage products, depending on the complement of processing enzymes found in the tissue or cell type. For example, the neuropeptide precursor proenkephalin can be processed into different sets of peptides by PC1/3, PC2 or furin [11]. Another classic example involves the differential processing of proopiomelanocortin (POMC). The anterior pituitary expresses only PC1/3 and cleaves POMC into adrenocorticotropin, whereas cells in the intermediate pituitary and neurons in the hypothalamus express PC2 which further cleaves adrenocorticotropin into two smaller peptides: α-melanocyte stimulating hormone and corticotropin-like intermediate lobe peptide [12]. Therefore, the peptides produced from a particular precursor are dictated by the cohort of enzymes produced by that cell and the internal environment of the vesicle which influences the activity of the enzymes.

It is generally thought that the various peptides produced from each precursor are packaged together into relatively homogenous secretory granules [13]. One exception was found for *Aplysia californica* egg-laying hormone, which appeared to be processed into two peptides that were sorted into distinct vesicles [14], [15]. This was also observed when egg-laying hormone was expressed in AtT-20 cells; the two peptides produced from this precursor were sorted to distinct populations of secretory granules [15]. Another example is the precursor to thyrotropin releasing hormone; the N- and C-termini of this precursor are sorted into different secretory vesicles [16]. Thus, mammalian cells have the ability to sort peptides derived from a single protein into different secretory vesicles. There are also examples in which different soluble proteins are sorted into distinct secretory granules [17].

**Figure 1:**
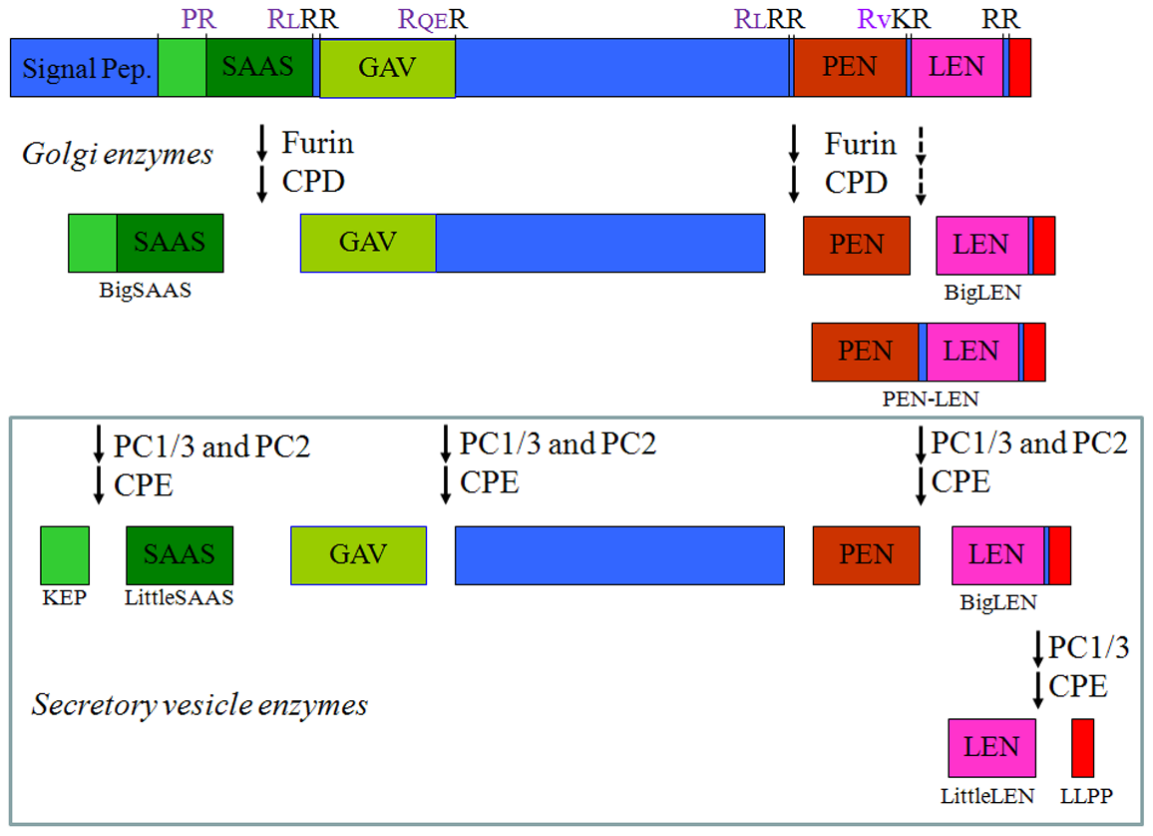
Schematic diagram of proSAAS and proSAAS-derived peptides. The major peptides derived from proSAAS are indicated, along with the enzymes involved in the cleavages. The amino acids within the cleavage site are shown (top row); residues in black are removed by the carboxypeptidase (CPD or CPE), and residues in purple remain on the C-terminus of the upstream peptide. The sites that are efficiently cleaved by furin/CPD are indicated by solid arrows; these cleavages generate big SAAS, an intermediate peptide representing GAV and the mid-portion of proSAAS, and PEN-LEN. Furin can also cleave PEN-LEN into PEN and big LEN (dashed arrow) but this cleavage does not go to completion in mouse brain. Within secretory vesicles, big SAAS is cleaved into KEP and little SAAS, the middle portion of proSAAS is cleaved into GAV and an un-named peptide, PEN-LEN is cleaved into PEN and big LEN, and big LEN is converted into little LEN and a 4-residue peptide LLPP. The conversion of big LEN into little LEN and LLPP is primarily catalyzed by PC1/3 and CPE. In addition to these major forms of the proSAAS-derived peptides, smaller forms of little SAAS, GAV, and PEN are present in mouse brain although the levels of these truncated peptides are generally lower than the levels of the peptides indicated in this figure.

In the present study, we examined the processing and sorting of the peptide precursor named proSAAS, which is cleaved into peptides named SAAS, GAV, PEN, and LEN (Figure 1) [18]. SAAS and LEN are found in big and little forms, depending on the extent of the processing. ProSAAS is broadly expressed in brain, with highest levels in the arcuate nucleus of the hypothalamus where it is colocalized with neuropeptide Y (NPY) [19]. ProSAAS transgenic mice and proSAAS knock-out mice studies revealed a body weight phenotype indicative of an orexigenic role for the peptides derived from proSAAS [20], [21]. Further studies revealed an orexigenic role for big LEN and PEN [19]. In this previous study, as well as in other studies [22], [23], it was noted that different proSAAS-derived peptides show distinct patterns of distribution. In the present study, the distribution of proSAAS-derived peptides was examined in mouse hypothalamus and in AtT-20 cells. The results of these studies suggest that peptides derived from proSAAS are differentially sorted to distinct vesicular populations.

## Materials and Methods

### Reagents

All reagents were obtained from Sigma-Aldrich (St. Louis, MO, USA) unless otherwise indicated. Rabbit antisera to big LEN (#85), SAAS (#2768) and PEN (#142) were previously generated [23]. Selectivity of the antisera for their peptides has been established, and cross-reactivity of these antisera with other proSAAS-derived peptides has been previously reported [22]. Importantly, the antiserum raised to big LEN displays less than 0.1% cross reactivity to little LEN while the antiserum raised to little SAAS cross reacts with big SAAS and cannot distinguish these two peptides [22]. Therefore, in the present study the term SAAS is used to refer to the results obtained with the antiserum raised to little SAAS peptide. PEN-LEN antiserum was generated in chicken and characterized [24]. Little LEN antiserum (#40), a gift from Dr. Iris Lindberg, was raised to the C-terminus of the peptide [25]. Under the conditions used for immunohistochemistry, the signal obtained with this antiserum is displaced by little LEN but not by big LEN, indicating the antiserum to be selective for the smaller form.

### Antibody purification and labeling

For the studies using antibodies directly labeled with fluorescent dye, Protein A agarose columns (Invitrogen, Carlsbad, CA, USA) were used to purify the IgG fraction of antibodies prior to labeling according to manufacturers' instructions. The IgG concentration of each eluate was determined using a spectrophotometer. IgG fractions of antibodies to big LEN and SAAS were labeled with Alexa 488 using the Microscale Protein labeling kit (Invitrogen, Carlsbad,CA) according to the manufacturer's instructions.

### Animals

Male C57BL/6J mice were obtained commercially (Jackson Laboratory, Bar Harbor, ME). NPY-GFP mice (B6.FVB-Tg(Npy-hrGFP)1Lowl/J) mice were the gift of Drs. Clemence Blouet and Gary Schwartz (Albert Einstein College of Medicine), and were originally obtained from Jackson Laboratory (Bar Harbor, ME). ProSAAS knock-out mice were the gift of Drs. Dan Morgan and John Pintar (Rutgers) [20]. All efforts were made to minimize the suffering and the number of animals used. This study was carried out in strict accordance with the recommendations in the Guide for the Care and Use of Laboratory Animals of the National Institutes of Health. The protocol was approved by the Institutional Animal Care and Use Committee and the Institute for Animal Studies of Albert Einstein College of Medicine (protocol number 20090305).

### Cell Culture

AtT-20 mouse pituitary corticotroph cells were grown in Dulbecco's modified Eagle's medium supplemented with 10% fetal bovine serum and penicillin/streptomycin (10 units/ml). AtT-20 cells stably expressing rat proSAAS were generated as previously described [18].

### Tissue preparation for immunohistochemistry

After anaesthetization using diethyl ether, mice were transcardially perfused with 4% phosphate buffered paraformaldehyde through the ascending aorta. Brains were removed and postfixed overnight at 4°C in 4% phosphate buffered paraformaldehyde. Brains were then dehydrated overnight using 30% sucrose in phosphate buffer. Brains were subsequently prepared for cutting by embedding in Tissue-Tek Optimal Cutting Temperature compound (Sakura Finetek, Torrance, CA, USA) and freezing in isopentane (Fisher Scientific Hampton, NH, USA) at −50°C and stored at −70°C until cutting. Sections were cut at 14 µm thickness on a sliding cryostat microtome from all tissues. Brains were cut in the region of the arcuate nucleus of the hypothalamus, from which all hypothalamic sections were taken. All tissues were thaw mounted onto Superfrost Plus microscope slides (Fisher Scientific, Hampton, NH, USA).

### Immunohistochemistry

Tissue sections were washed on slides with phosphate buffered saline (PBS), then blocked for 2 hours at room temperature in 5% bovine serum albumin (BSA) in PBS containing 0.5% Triton X-100 in a humidity chamber to prevent drying of blocking solution. Slides were then incubated for 24 hours at 4°C with either chicken anti-PEN-LEN (1∶20,000) and/or 1∶10,000 dilutions of rabbit antisera to big LEN, PEN, SAAS, or little LEN in 5% BSA in PBS with 0.5% Triton X-100. After washing with PBS containing 0.2% Tween-20, secondary Cy3- and Cy2-conjugated antibodies (Jackson Immunoresearch, West Grove, PA, USA) were applied. For double labeling using Alexa 488 directly conjugated primary antibody IgG fraction, slides were first incubated with the unlabeled primary antiserum and secondary antiserum (described above) and after washing were then incubated overnight with labeled purified IgG fraction of antibodies to big LEN or SAAS. After overnight incubation at 4°C, the sections were washed and mounted in anti-fade reagent containing 4,6- diamidino-2-phenylindole (DAPI; Invitrogen, Carlsbad, CA, USA). Pre-immune serum from each of the animals used for the proSAAS-derived peptide antisera were applied to adjacent sections at the same dilutions as the antisera to control for non-selective staining. Previous studies in our lab controlled for antibody selectivity by pre-incubating antibodies with their respective peptides prior to application to tissues [19].

### Immunocytochemistry

For immunocytochemical staining of AtT-20 cells stably expressing rat proSAAS, cells were first plated on polylysine coated coverslips in 12 well dishes (Corning, Corning, NY, USA). Cells were fixed at low confluence using 4% paraformaldehyde in PBS. Following fixation, cells were rinsed repeatedly with phosphate buffer and then permeablized with 0.1% Triton X-100 detergent in PBS. Cells were then blocked using 4% bovine serum albumin (BSA) in PBS for one hour. Primary antibodies to big LEN, little LEN, SAAS, PEN-LEN, or PEN were incubated with cells in BSA/PBS for one hour. Cells were rinsed with PBS and incubated for one hour with anti-chicken and/or anti-rabbit Cy3 and Cy2 conjugated secondary antibodies (Jackson Immunoresearch, West Grove, PA). For experiments using directly labeled primary antibodies, incubation was carried out for one hour, cells were rinsed thoroughly with PBS and affixed to microscope slides using Prolong Gold reagent anti-fade reagent containing 4,6- diamidino-2-phenylindole (DAPI; Invitrogen, Carlsbad, CA, USA).

### Site-directed mutagenesis

Mutant forms of rat proSAAS were generated via site-directed mutagenesis using the Quikchange Site-Directed Mutagenesis kit (Stratagene) according to the manufacturers' instructions. Briefly, pcDNA3.1 (-) (Invitrogen, Carlsbad, CA) containing the rat proSAAS cDNA sequence was mutated using PCR mutagenesis with specific primers designed to convert the two RLRR furin consensus sites (Figure 1) into prohormone convertase consensus sites (KLRR); furin is optimal with Arg in the -4 position while PC1/3 and PC2 do not need this upstream Arg if the P1 and P2 are both basic residues. The resulting plasmid with Arg58Lys and Arg217Lys was confirmed by nucleotide sequencing. AtT-20 cells were transiently transfected with either wild type proSAAS or mutant proSAAS plasmids.

### Image Analysis

All images were processed using Photoshop (Adobe Systems Inc.) and ImageJ (http://imagej.nih.gov) and analyzed using the JACoP plugin for ImageJ (http://rsb.info.nih.gov/ij/plugins/track/jacop.html).

## Results

### ProSAAS-derived peptides in mouse brain

**Figure 2:**
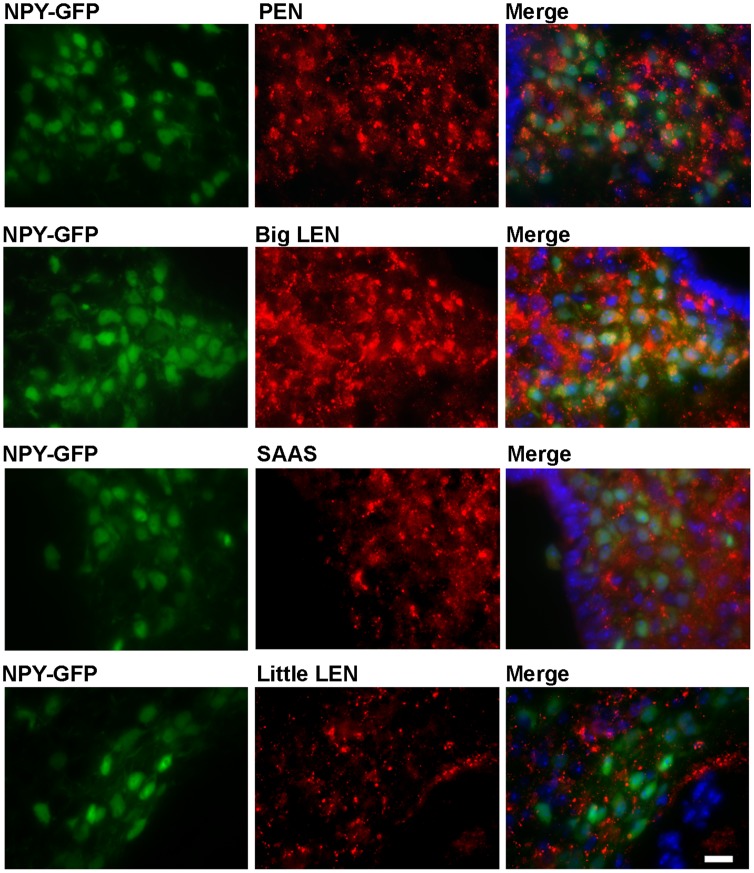
Comparison of the distribution of proSAAS-derived peptides and NPY-expressing cells in the arcuate nucleus of the hypothalamus. Immunofluorescence was performed in mice expressing GFP under the NPY promoter, as described in Materials and Methods. Left panels show the distribution of GFP, which is localized to the cytosol and reveals the cell bodies of NPY-positive neurons. Middle panels show immunofluorescence of the indicated proSAAS-derived peptide. Right panels show the merged images from the left and right panels. Cell nuclei were visualized with DAPI (blue color, right panels). Scale bar = 10 µm.

ProSAAS-derived peptides and NPY are among the most abundant peptides in the hypothalamus [1],[26]. In a previous study using immunofluorescence, PEN-LEN and NPY showed substantial colocalization in the arcuate nucleus of the hypothalamus, with a punctate pattern suggesting localization to the vesicles of the secretory pathway of the neurons. In addition, big LEN and PEN localize to neurons in the arcuate nucleus expressing GFP under the NPY promoter [19]. In these mice, GFP is expressed as a cytoplasmic protein, not within the secretory pathway, allowing visualization of the entire NPY-expressing cell body [27]. Colocalization is evident in the merged images by the appearance of yellow punctate staining within a green cell body (Figure 2, right panels). In contrast to the results with either PEN or big LEN, both of which show substantial colocalization with GFP-expressing cells, neither SAAS nor little LEN showed substantial overlap with the GFP expression, indicating these peptides are largely absent from cell bodies of NPY-expressing cells (Figure 2).

**Figure 3:**
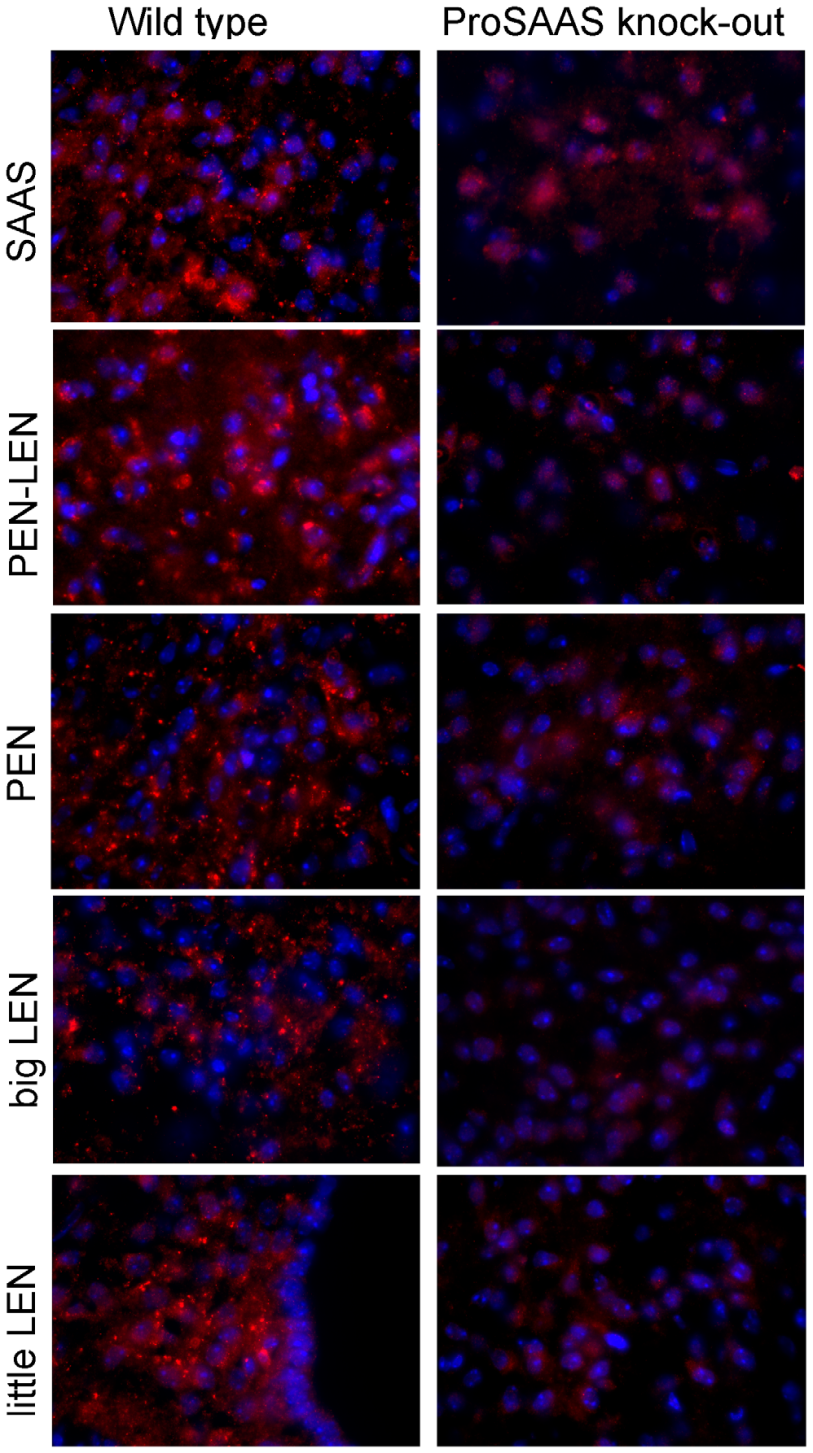
Comparison of proSAAS-derived peptide immunofluorescence in the arcuate nucleus of the hypothalamus in wild type and proSAAS knock-out mice. Immunofluorescence was performed as described in Materials and Methods, using a Cy3-labeled secondary antiserum (red). Cell nuclei were visualized with DAPI (blue). Antisera to SAAS, PEN-LEN, PEN, big LEN, and little LEN show a strong punctate staining pattern in wild type mouse hypothalamus (left panels) which is absent in the knock-out mouse brain (right panels).

Previous studies tested the selectivity of the antibodies using blocking controls [19]. Because blocking controls only show that the epitope is recognized by the antibodies, we performed additional controls using proSAAS knock-out mouse brain; the generation of proSAAS knock-out mice has been recently reported [20]. Antibodies to SAAS, PEN-LEN, PEN, big LEN, and little LEN showed a punctate pattern in wild type mouse hypothalamus (Figure 3, left panels). Some of the antibodies (i.e. SAAS) showed a diffuse background staining in the knock-out mouse brain section. However, the strong punctate staining seen in the wild type mouse brain was absent in the knock-out mouse brain (Figure 3, right panels).

**Figure 4:**
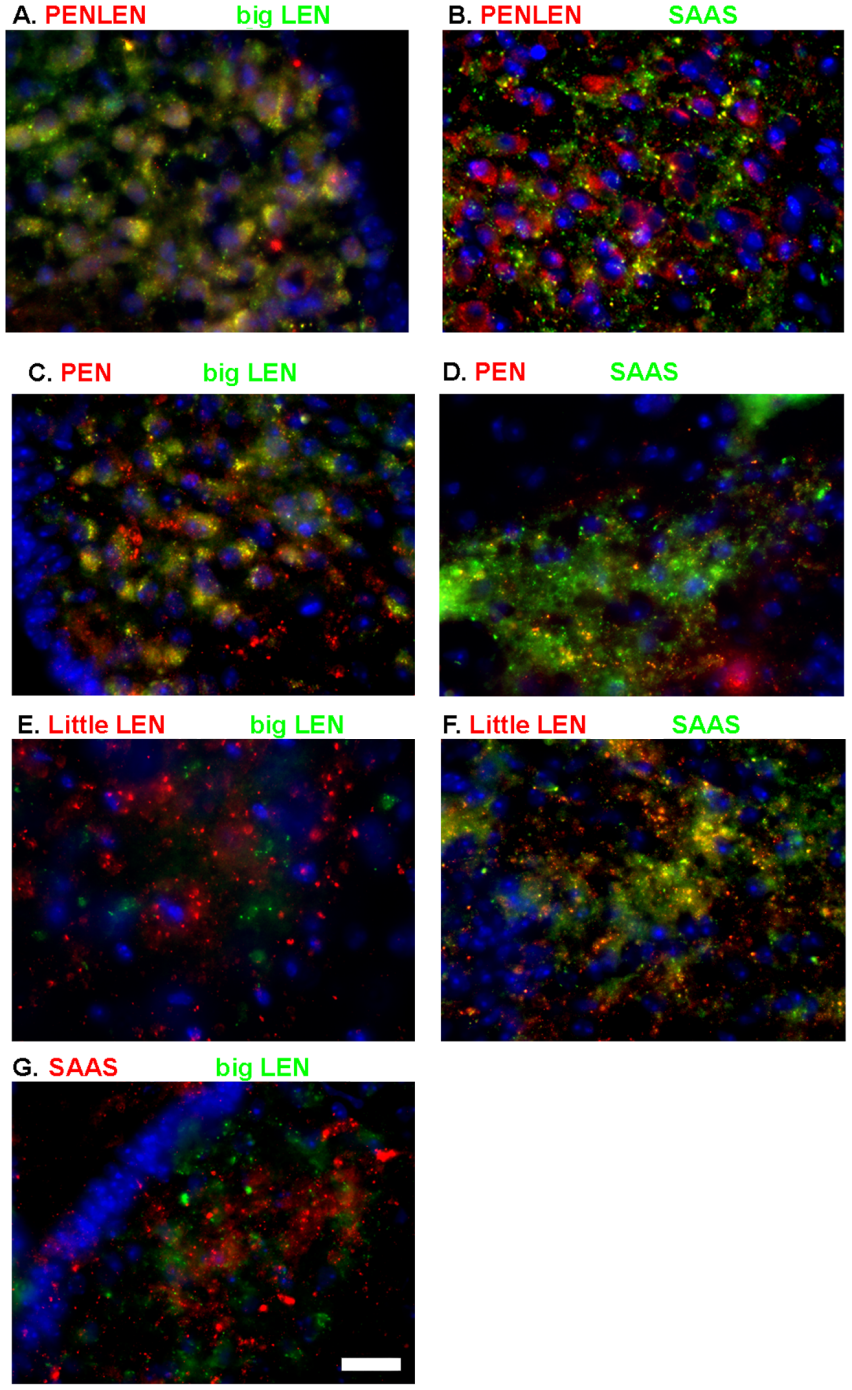
Localization of proSAAS-derived peptides in the arcuate nucleus. Immunofluorescence was performed as described in Materials and Methods using antibodies directly labeled with Alexa 488 (big LEN and SAAS), or with unlabeled primary antisera followed by Cy2- or Cy3-labeled anti-rabbit or anti-chicken secondary antibodies. A: PEN-LEN (in red, Cy3) and big LEN (in green, Cy2). B: PEN-LEN (Cy3) and SAAS (Cy2). C: PEN (Cy3) and big LEN (Alexa 488 directly labeled). D: PEN (Cy3) and SAAS (Alexa 488 directly labeled). E: Little LEN (Cy3) and big LEN (Alexa 488 directly labeled). F: Little LEN (Cy3) and SAAS (Alexa 488 directly labeled). G: SAAS (Cy3) and big LEN (Alexa 488 directly labeled). Scale bar = 10 µm.

The colocalization of various proSAAS-derived peptides was further examined in the arcuate nucleus of wild type mice. PEN-LEN shows strong colocalization with big LEN but not with SAAS (Figure 4A, B). Similarly, PEN and big LEN showed extensive overlap, but not PEN and SAAS (Figure 4C, D). Big and little LEN did not show overlap (Figure 4E), even though these two peptides are derived from the same region of proSAAS (Figure 1). Some colocalization of little LEN and SAAS was observed (Figure 4F) but this appeared to be less extensive than colocalization of PEN-LEN with big LEN (Figure 4A) or PEN with big LEN (Figure 4C). SAAS and big LEN showed negligible overlap (Figure 4G). Taken together, these results indicate that some, but not all of the proSAAS-derived peptides colocalize with each other.

### ProSAAS-derived peptides in AtT-20 cells

**Figure 5:**
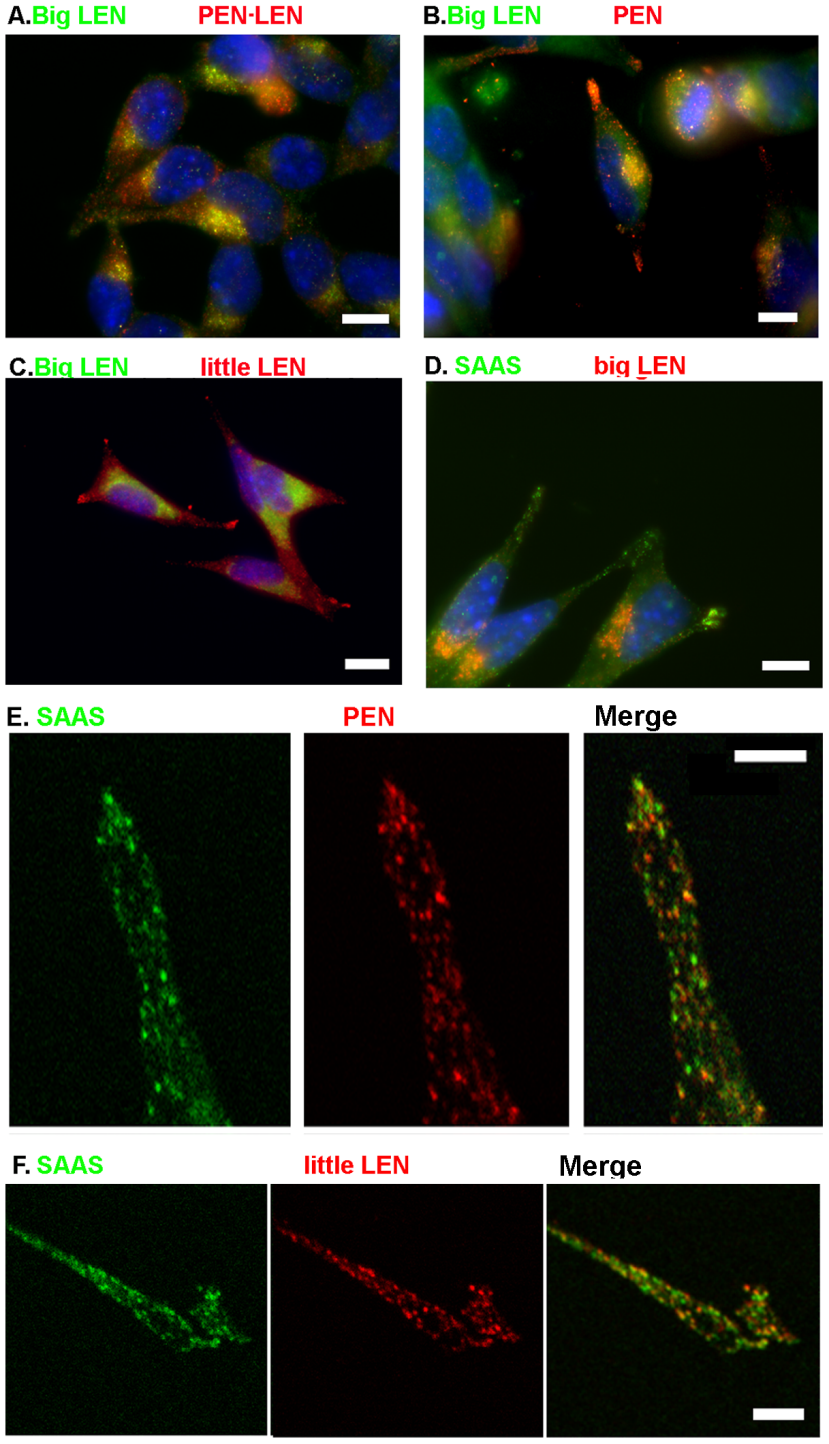
Localization of proSAAS-derived peptides in AtT-20 cells. Immunofluorescence was performed as described in Materials and Methods. A: Big LEN (Cy2) and PEN-LEN (Cy3). B: Big LEN (Alexa 488 directly labeled) and PEN (cy3). C: Big LEN (Alexa 488 directly labeled) and little LEN (Cy3). D: SAAS (Alexa 488 directly labeled) and big LEN (Cy3). E: SAAS (Alexa 488 directly labeled, left panel) and PEN (Cy3, middle panel). The right panel is a merge of the two images. F. SAAS (Alexa 488 directly labeled, left panel) and little LEN (Cy3, middle panel). Right panel is a merge. Scale bars in panels A, B, C, and D indicate 10 µm. Scale bars in panels E and F represent 1 µm.

The distribution of the proSAAS-derived peptides observed in the arcuate nucleus could be due to a number of factors, including differential sorting of the proSAAS-derived peptides into distinct vesicles and/or differential cleavage of proSAAS by the various processing enzymes. AtT-20 mouse pituitary corticotroph cells overexpressing proSAAS [24], [28] allow the observation of proSAAS-derived peptides in a uniform population of cells expressing the same processing enzymes. Furthermore, the use of cell culture allows for the detection of individual cells and the visualization of subcellular compartments. Specifically, mature secretory vesicles are found at the tips of AtT-20 cell processes whereas the immature vesicles, TGN, and Golgi show a perinuclear distribution in these cells [29]. The directly-labeled IgG fractions of antibodies to big LEN and SAAS were employed for immunocytochemical staining of AtT-20 cells, in combination with antisera to the other proSAAS-derived peptides. Big LEN and PEN-LEN both show intense immunolocalization to the perinuclear space in the AtT-20 cells and are generally absent from the tips of cells (Figure 5A); this distribution indicates that these peptides are present in Golgi, TGN, and/or immature vesicles and are not present in mature vesicles, presumably because they are further processed into smaller peptides in the mature vesicles. In contrast, PEN immunofluorescence is present in both the perinuclear space and tips of cell processes, and only overlaps with big LEN in the perinuclear space (Figure 5B). Little LEN shows intense immunofluorescence in the tips of the cells and only weak staining of the perinuclear area (Figure 5C), consistent with the processing of big LEN into little LEN by PC1/3 in the secretory vesicles. SAAS also shows intense staining in the tips of the AtT-20 cells (Figure 5D). Co-staining with big LEN shows little colocalization of this peptide and either little LEN or SAAS in AtT-20 cells (Figure 5), similar to the lack of colocalization of big LEN and these peptides in the hypothalamus.

With the observation that PEN, little LEN and SAAS all show a vesicular staining pattern in the tips of the AtT-20 cells, comparison of these peptides' staining patterns with one another is critical to determine if the peptides all localize to the same vesicles as processed peptides, or whether they are sorted to different vesicle populations. For this, confocal microscopy was used to provide a high level of resolution. Comparisons of SAAS and PEN show some colocalization of these peptides in the tips of AtT-20 cells, indicated by yellow punctae in the merged images (Figure 5E). However, there are many vesicles that appear to be mainly red or green, suggesting populations of vesicles that contain predominately either SAAS or PEN. Similar analysis comparing SAAS and little LEN produced a similar result, with some vesicles containing both peptides but many vesicles containing predominantly one of the two peptides (Figure 5F).

### Site directed mutagenesis of furin-like cleavage sites

ProSAAS contains three sites with the consensus RxRR or RxKR (Figure 1); all three were previously found to be cleaved upon extended incubation of proSAAS with furin [25]. However, only two of these sites were cleaved upon shorter incubations with furin; the RxKR site separating PEN and LEN (Figure 1) was not cleaved under these conditions [25]. This matches the predictions of a computer program that uses an ensemble of neural networks to predict furin and prohormone convertase sites within proteins [30]. This program, available at http://www.cbs.dtu.dk/services/ProP/, ranks sites by probability of cleavage on a scale from 0 to 1, and a score above 0.5 is likely to be cleaved. According to this program, the first and second sites that are efficiently cleaved by furin [25] have scores of 0.714 and 0.818 while the third inefficient furin site has a score of 0.325. Cleavage of proSAAS at either of the efficient furin consensus sites separates the SAAS-containing region from the PEN and LEN-containing region of the precursor, and it is possible that these fragments are then sorted into different populations of vesicles for further processing into the mature forms of the peptides. To test this, we mutated the RLRR sequence in the two efficient furin consensus sites into KLRR; furin prefers RxRR whereas both PC1/3 and PC2 readily cleave at KxRR sites [2]. The neural network furin consensus site prediction program scores the double KxRR mutant below the threshold for efficient cleavage by furin or furin-like enzymes (values of 0.096 and 0.461 for sites 1 and 2). However, both sites are still favorable for cleavage by PC1/3 and/or PC2, with probability scores of 0.659 and 0.938 for sites 1 and 2. As a side point, PC1/3 is the predominant prohormone convertase in AtT-20 cells, and PC2 is either absent or present at low levels [31].

**Figure 6:**
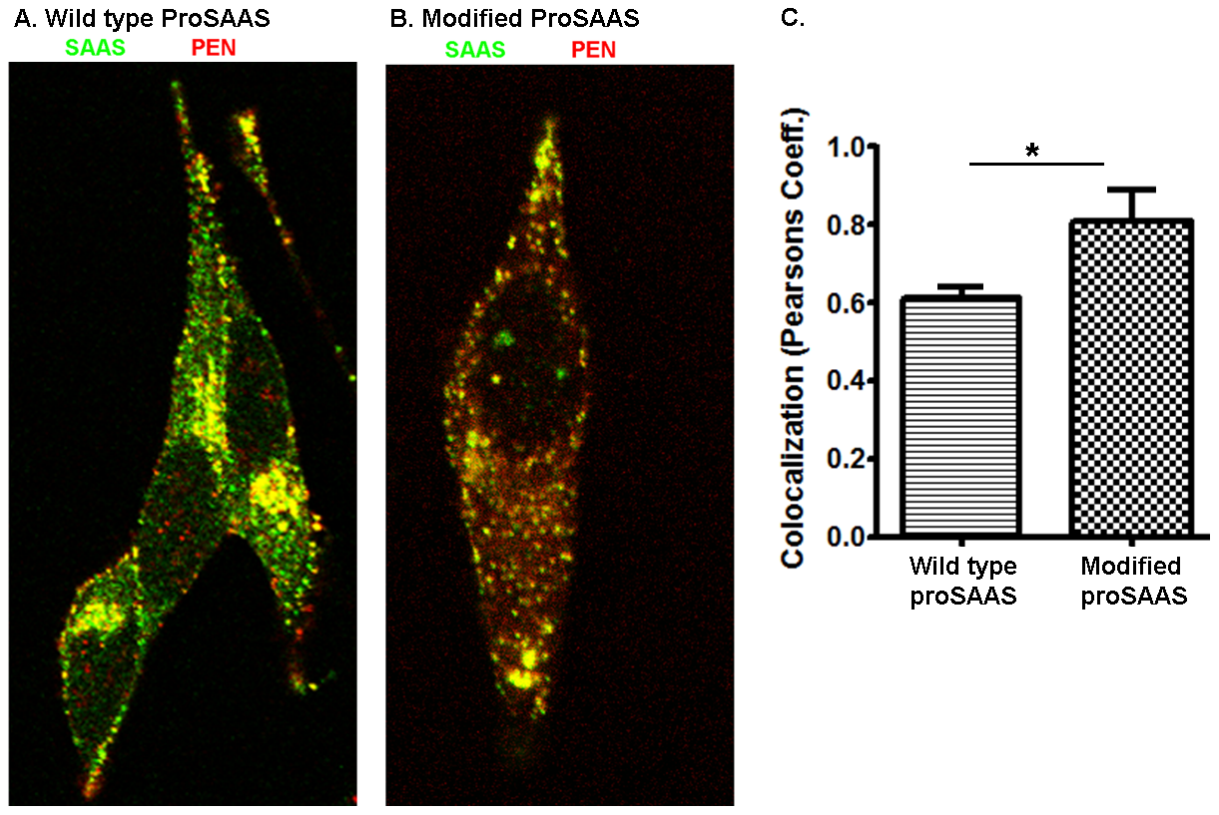
Effect of mutation of the two major furin cleavage sites on the localization of SAAS and PEN in AtT-20 cells. Wild type rat proSAAS was transiently expressed in AtT20 cells and peptides examined by immunofluorescence as described in Materials and Methods using antiserum to PEN (Cy3) and SAAS (Alexa 488 directly labeled). A: Wild type rat proSAAS. B: Rat proSAAS with the two major furin sites (RxRR) mutated to KxRR, which eliminates the furin consensus site. C. Quantification of colocalization as measured by Pearson's Coefficient. At least 30 cells were averaged per condition. Error bars represent standard error of the mean. *, p<0.05 using Student's t-test.

The mutant forms of proSAAS were transiently expressed in AtT-20 cells and the cells were co-stained for SAAS and PEN. Whereas the transiently expressed wild type rat proSAAS showed only partial overlap of SAAS and PEN (Figure 6A), proSAAS with the mutated furin consensus sites showed much more overlap of these two peptides (Figure 6B). Analysis using Pearsons coefficient of colocalization showed a significant difference in the overlap of PEN and SAAS in AtT-20 cells when comparing the wild type and mutant proSAAS (Figure 6C).

## Discussion

The major finding of these studies is that the proSAAS-derived peptides are differentially localized in brain. Localization of a neuropeptide is a strong indicator of its function, with many neuropeptides involved in feeding and body weight regulation localizing to the hypothalamus, particularly in the arcuate nucleus [32]. Previous studies have shown that proSAAS-derived peptides are enriched in this area [19], [23], and that in these brain areas the proSAAS-derived peptides PEN and big LEN colocalize with NPY, an established body weight-regulating peptide. Colocalization of neuropeptides with one another has been shown to be an important indicator of shared or synergistic function [33]. In the present study, it was discovered that not all of the proSAAS-derived neuropeptides localized to the NPY expressing neuronal cell bodies. Little LEN and SAAS are not seen in NPY neuronal cell bodies and also do not colocalize with either PEN-LEN or big LEN. This evidence of differential localization appears to be functionally relevant, as those peptides which colocalize with NPY (big LEN and PEN) appear to affect feeding while the peptides which do not colocalize with NPY (little LEN and SAAS) do not seem to influence feeding, based on studies examining the effect of neutralizing antibodies directed against the peptides [19]. Instead of a role in feeding, SAAS has been shown to play a role in circadian rhythm [34] and may have other functions.

One reason for the differential distribution of proSAAS peptides in brain is likely to be the result of processing of the precursor into distinct peptides. The processing of proSAAS is known to involve a number of enzymes [25], [28], [35]–[37]. Initial studies in AtT-20 cells found that newly synthesized proSAAS was cleaved at sites near the N- and C-termini within 20 minutes of the addition of radiolabeled amino acids, consistent with the action of furin or other Golgi/TGN enzymes [18]. ProSAAS contains three furin consensus sites (Arg-Xaa-Lys/Arg-Arg, where Xaa is any amino acid), and all three were found to be cleaved when bacterially-expressed proSAAS was incubated for long time periods with furin [25]. Only two of these sites were cleaved upon shorter periods of incubation with furin; the site between SAAS and GAV, and the site separating PEN-LEN from the middle region (Figure 1). When proSAAS was expressed in the PC12 cell line, which contains furin but not PC1/3 or PC2, the proSAAS was found to be cleaved into big LEN, PEN, and big SAAS, but not little LEN [28]. Peptidomics analysis of peptides in brains of *Cpe^fat/fat^* mice, which lack CPE activity, is useful to determine the extent of peptide processing by Golgi/TGN versus secretory pathway enzymes; CPE is only active within the acidic environment of the secretory vesicles and essentially inactive at the neutral pH of the Golgi while CPD functions in this compartment and in immature vesicles but is excluded from the mature granules [38]. Peptides such as GAV and little LEN are reduced by more than 80–90% in *Cpe^fat/fat^* mouse brain, relative to levels in wild type brain, consistent with a major role for CPE, and by inference, PC1/3 and/or PC2 and not furin [35]. Analysis of mice lacking PC2 also shows that levels of GAV are ∼50 to ∼90% of wild-type levels, depending on the brain region, indicating that either PC1/3 or PC2 can perform this cleavage [39]. Levels of little LEN are decreased and big LEN is elevated in PC1/3 defective mice, and not PC2 defective mice, indicating that PC1/3 is the primary endopeptidase that cleaves big LEN into little LEN. The finding that big SAAS is present in *Cpe^fat/fat^* mouse brain at levels comparable to those in wild type mouse brain [35] indicates that CPE is not involved in the production of SAAS, implying that furin or other Golgi/TGN endopeptidases followed by CPD are the main enzymes. Levels of PEN are partially reduced by 30–40% in *Cpe^fat/fat^* mouse brain [35], suggesting that cleavage of PEN-LEN into PEN is mediated in part by furin and CPD and in part by PC1/3 and/or PC2 together with CPE. Taken together, these results indicate that furin efficiently cleaves the sites indicated by solid arrows in Figure 1, and only partially cleaves the site indicated by a dashed arrow in Figure 1. It is possible that the degree of processing of the proSAAS peptides by furin-like enzymes in the TGN bears some responsibility for the differential localization of the mature proSAAS-derived peptides. Recent studies suggest that regulation of furin is possible in the TGN, and this could contribute to particular proSAAS peptides being cleaved or not, depending on the cell-specific regulation of TGN processing by furin [40].

The cleavage site required to process big LEN into little LEN is not a furin site. Furthermore, neither furin nor PC2 were found to cleave bacterially-expressed proSAAS into little LEN [25]. Peptidomics of mice lacking PC1/3 activity showed a decrease in levels of little LEN and an increase in levels of big LEN in various brain regions, suggesting that PC1/3 is the major enzyme converting big LEN into little LEN in brain [41]. Similar analysis of mice lacking PC2 did not show a change in levels of big LEN [36], [37]. Collectively, these studies indicate that the conversion of big LEN into little LEN is mainly carried out in brain by PC1/3, and not PC2. Therefore, the differential distribution of big and little LEN in brain may be partially a result of the variable expression of PC1/3; cells that have high levels of PC1/3 would convert big LEN into little LEN, while cells with low levels of PC1/3 would mainly contain big LEN. However, this would not explain our finding that big LEN, and not little LEN, is detected in the NPY-expressing cell bodies (Figure 2). ProNPY is more efficiently processed into NPY by PC1/3 than by PC2 (together with CPE and the amidating enzymes), based on in vitro kinetic studies [42]. Furthermore, PC2 knock-out mice show only a modest reduction in levels of NPY, consistent with a major role for PC1/3 and not PC2 [36], [37].Therefore, a more likely explanation to account for the lack of little LEN in NPY-expressing cell bodies is that the conversion of big LEN to little LEN occurs later in the secretory pathway, after the secretory vesicles have left the cell body. The studies on AtT-20 cells (Figure 5) support this model, with big LEN detected in the perinuclear region and little LEN primarily found in the axonal tips. PEN is also found in the tips of AtT-20 cells, as well as the perinuclear region, consistent with the previous studies that found PEN to be partially produced by furin (together with CPD). The antiserum to SAAS, which detects both big and little SAAS, also shows both a perinuclear and tip localization, consistent with the furin/CPD mediated processing of proSAAS into SAAS described in the above studies.

Although there is some colocalization of SAAS and PEN in AtT-20 cells, distinct populations of vesicles are enriched in one of these two peptides (Figures 5 and 6). These results cannot be explained by differential processing because both antisera recognize the products of the Golgi/TGN enzymes (furin, CPD) as well as the products of the secretory vesicle enzymes (PC1/3, PC2, and CPE). An alternative explanation is that the products of the Golgi/TGN enzymes are sorted into distinct populations of vesicles, containing either the N-terminal region (big SAAS) or the C-terminal region (PEN-LEN, PEN, big LEN). Once sorted, PC1/3 and CPE in the secretory granules of AtT-20 cells convert these peptides into the smaller products (KEP, little SAAS, little LEN, and LLPP). Evidence in support of this model was provided by the mutation of the two major furin sites in proSAAS (Figure 1), expression in AtT-20 cells, and comparison of the colocalization of SAAS and PEN. By converting furin consensus sites (R-X-K/R-R) into PC1/3 or PC2 consensus sites (K-X-K/R-R), the processing was delayed until proSAAS reached the immature secretory granule where PC1/3 and PC2 are active. The finding that SAAS and PEN showed significantly more colocalization when the furin sites were mutated (Figure 6) supports the hypothesis that the N- and C-terminal regions of proSAAS are cleaved by furin and sorted into distinct vesicles.

There are several other examples in which individual peptides derived from a single precursor are sorted into distinct vesicles. One previous study found that expression of prothyrotropin-releasing hormone in AtT-20 cells resulted in peptides that were sorted into different secretory granules, and another study found a similar result with neurotrophins [43], [44]. Sorting in this manner has also previously been shown to occur with *Aplysia* peptides expressed in AtT-20 cells [15]. It was proposed that self-aggregation of the protein/peptide components is an important factor in their sorting into granules [17]. Sorting of proSAAS-derived peptides into distinct populations of granules has important implications for the regulation and function of the proSAAS-derived peptides. It has previously been shown that all proSAAS-derived peptides do not carry out the same function in the mouse brain [19]. Distributing the different peptides to various sets of vesicles could distinguish functional peptides from one another. These vesicles could therefore release their contents in distinct brain regions and affect different downstream responses in the mouse brain.
